# Feasibility Study for Monitoring an Ultrasonic System Using Structurally Integrated Piezoceramics

**DOI:** 10.3390/s24031036

**Published:** 2024-02-05

**Authors:** Jonas M. Werner, Tim Krüger, Welf-Guntram Drossel

**Affiliations:** 1 Professorship for Adaptronics and Lightweight Design in Production, Chemnitz University of Technology, Reichenhainer Straße 70, 09126 Chemnitz, Germany; tim.krueger@s2015.tu-chemnitz.de (T.K.);; 2 Fraunhofer Institute for Machine Tools and Forming Technology IWU, 09126 Chemnitz, Germany

**Keywords:** ultrasonics, piezoelectric sensors, monitoring

## Abstract

This paper presents a new approach to monitoring ultrasonic systems using structurally integrated piezoceramics. These are integrated into the sonotrode at different points and with different orientations. The procedure for integrating the piezoceramics into the sonotrode and their performance is experimentally investigated. We examine whether the measured signal can be used to determine the optimal operating frequency of the ultrasonic system, if integrating several piezoceramics enables discernment of the current vibration shape, and if the piezoceramics can withstand the high strains caused by the vibrations in a frequency range of approximately 20–25 kHz. The signals from the piezoceramic sensors are compared to the real-time displacement at different points of the sonotrode using a 3D laser scanning vibrometer. To evaluate the performance of the sensors, different kinds of excitation of the ultrasonic system are chosen.

## 1. Introduction

Higher standards of precision and productivity drive the continuous development of machine tools. Higher performance and functionality are achieved by integrating mechatronic systems [1]. Ultrasonic systems are one kind of these systems and can provide a performance increase in different ways [2]. These systems are used in a variety of processes, like welding, where the vibrations can improve material mixing and uniform grain growth and thereby increase the strength of the connection [3]. In deep drawing processes, the maximum drawing ratio can be increased by using an ultrasonic vibration-assisted process. This is achieved by decreasing the effective process force [4]. In machining processes, e.g., turning, ultrasonic assistance can be used to extend tool life and enable the machining of harder-to-machine materials. Additionally, the chip size, surface roughness and burr formation can be affected positively by using ultrasonic-assisted turning [5]. The mechanics behind these effects have been thoroughly investigated. The reasons stated are a shorter contact time between the tool and workpiece and, therefore, a shorter time for diffusion processes, decreased cutting forces and better chip breaking. Research focused on using ultrasonic vibration for generating specific surfaces and certain surface properties has been published [6,7]. The goal of these studies was the manufacturing of microstructures on the workpiece by using ultrasonic vibration in a radial direction to the sample during turning. Manufacturing microstructures in one process step is a highly productive process. Zhang et al. [8] proposed a two-stage process, where dimples are generated on a surface. First, a microstructured surface is machined by using ultrasonic vibration-assisted turning. In the second step, the peaks of the surface are removed by using conventional turning with a very small depth of cut. Processes like these are heavily reliant on an efficient control system of the ultrasonic system to achieve advantages, like the generation of geometrically defined microstructures by cutting. This is especially crucial regarding the result of the manufacturing process. A control system for these ultrasonic systems is necessary to obtain a constant vibration amplitude at the desired frequency. Currently, this is usually carried out by an in-process measurement of electrical parameters inside the actuator and therefore, relatively far away from the cutting process. The operating frequencies start upwards of 18 kHz and achieve vibration amplitudes of approx. 20 μm peak-to-peak [5]. Therefore, the system consisting of a transducer, a sonotrode and a generator also usually incorporates a controller [2]. The generator supplies power to the transducer, which converts the electrical energy into a mechanical vibration at the resonance frequency of the system. The vibrations are generated using the indirect piezoelectric effect of piezoelectric discs. Two masses at the ends of the transducer are used for tuning the system and a bolt is used for preloading the piezoelectric disc to protect them from damage during operation. If the vibration amplitude generated by the transducer is not enough for the specific process, a sonotrode can be used to amplify the vibration using a geometrical transformation. In most cases, a longitudinal vibration shape of the system is desired. For this the transducer and sonotrode expand and contract, which causes areas of high strain and zero displacement, called nodes, and areas of zero strain and high displacement, called antinodes. Since there is no displacement at the nodes, these are used for mounting the system. Furthermore, the cutting tool is placed at the end of the sonotrode. The highest amplitudes can only be achieved if the excitation frequency of the generator equals the natural frequency of the system, which comprises one of the disadvantages of these systems. Therefore, to generate predefined microstructures, a control system for controlling the amplitude as well as the excitation frequency is necessary. Using a fixed excitation frequency is suboptimal: because of external conditions, such as a changing load or thermal influences, the natural resonance frequency of the system may exhibit a frequency shift [9]. Yokozawa states that the reasons for this may be non-linear effects [10]. Different algorithms for the control of ultrasonic systems have been proposed in the literature. Babitsky and Voronina have published the use of an “autoresonant control” that uses a feedback signal for closed-loop control [11,12]. Another commonly used system is “phase-locked loop” control. The goal of this control algorithm is to keep the phase shift between the supplied current and voltage at 0 degrees [13,14]. In theory, both internal electrical and external mechanical feedback signals may be used [15]. Electrical signals are usually used, since they are already available. Because of the physical distance between the electrical parts and the cutting tool and non-linear effects, using these signals may not be the best course of action. Mechanical signals offer a greater benefit regarding the content of information. Depending on the measured signal, a direct conclusion regarding the current vibration amplitude may be drawn. Babitsky also proposes that using mechanical signals allows for a better detection of the current vibration mode [15]. For this the displacement at the tool or the tip of the sonotrode, the vibration velocity and acceleration as well as the strain of the system are possible values. The limiting factors regarding the measurement of mechanical dimensions are the permanent fixation of sensors under industrial standards and, depending on the sensor, the optical accessibility. Babitsky and Voronina compared electrical and mechanical feedback in a simulation and showed that the maximum deviation of the root mean square is lower when using a mechanical signal closer to the cutting edge [15], which was confirmed in experimental investigations by Li using an inductive sensor in ultrasonic vibration-assisted drilling [16]. Previous work conducted by the authors investigated using strain gauges for monitoring an ultrasonic-assisted turning process. The results showed that using strain gauges enables the detection of the current vibration shape of the system and allows for in-process measurement of ultrasonic systems, which may be used in control systems in the future [17]. The ultrasonic system used by Kimme et al. also included an additional piezoelectric disc in the transducer to monitor the vibration and control the excitation frequency. Yokozawa et al. [10] proposed the same. Yu et al. proposed a self-sensing monitoring system for resonant control of an ultrasonic system used for cutting, but it uses the piezoelectric ceramics as well and not a sensor close to the cutting tool [18]. Much research has been performed on the monitoring of machine tools and mechanical systems. Teti et al. present the high importance of sensor systems and possibilities of close-to-process piezoelectric sensors [19]. Furthermore, different uses of integrated sensors are presented in [20,21,22]. Eiras et al. present an approach for monitoring aircraft health using bonded piezoelectric wafer sensors, while Khattak et al. developed a polyvinylidene flouride (PVDF) sensor that is embedded in a metal structure and can achieve a high linearity with this approach. Using a piezoelectric material may be an advantage over using strain gauges. Werner et al. compared strain gauges, piezoelectric patches as well as structurally integrated piezoceramics (SIP) regarding their suitability for measuring vibrations at different frequencies [23]. The results show that the SIPs exhibited a better signal quality at higher frequencies, since they have a higher sensitivity and a lower damping of the contact zone in comparison with the strain gauges, since the SIPs are applied to the structure by forming. A disadvantage of these SIPs is that they are not directly purchasable as sensors and it is not known yet if they can withstand the high strain of ultrasonic systems at high operating frequencies. Ultrasonic vibration-assisted machining offers many benefits, but to make optimum use of these, a control system with high precision is necessary. Control systems using mechanical signals might offer benefits over electrical signals, but only a few studies about this exist.

## 2. Materials and Methods

### 2.1. Structurally Integrated Piezoceramic

The sensor principle used in this work is based on the structural integration of piezoceramic elements with a rectangular cross-section into a workpiece by forming [24,25]. First, a cavity in the workpiece, in this case a sonotrode, is formed by milling. A punch is then used to form another piece of metal and thereby clamp the piezoceramic elements in the structure. A direct electromechanical coupling can therefore be achieved without the use of adhesives. In previous studies, these sensors have been integrated into materials like aluminum alloy EN AW-5083 [26,27] and steel sheets of X5CrNi18-10 [23]. In this paper, an approach to integrate them into a sonotrode made of Ti6Al4V is presented.

**Figure 1:**
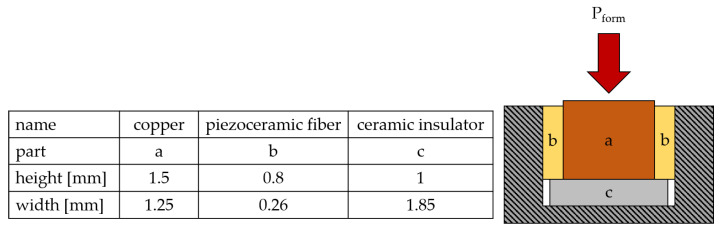
Manufacturing process for the integration of the piezoceramics and their dimensions.

The process used for forming is shown in Figure 1. A ceramic insulator is placed at the bottom of the cavity. Two piezo fibers and a wire are placed on top of it. An assembly gap of 0.13 mm enables assembly by hand. A hydraulic machine vise (Hilma KNC 100 by STARK Spannsysteme, Rankwell, Austria) is used to apply the load on the wire via a flat die and thereby deform the copper. The resulting forces in the cavity lock the piezoceramics in place and preload them. The preload force has to be chosen carefully in order not to damage the brittle piezoceramic fibers. If it is too high, the piezoceramics might break; if it is too low, the deformation will not suffice for an appropriate connection between the materials. Initial experiments show that an optimum forming load is $P_{form}$ = 390–470 N/${\mathrm{mm}}^{2}$. The piezoceramics showed no damage in an optical inspection using a microscope and the connection was not detachable by hand. Whether the connection can withstand the higher strains and vibrations of the ultrasonic system needs to be evaluated. The fibers are made from M1100 piezoceramic plates from Johnson Matthey [28]. These are available in different dimensions and therefore, usable for different applications. While the piezoelectric discs of the actuator use the inverse piezoelectric effect to generate vibrations, the piezoelectric plates use the direct piezoelectric effect, which means that they generate an electrical voltage when a mechanical load is applied. This enables their use as a sensor. The piezoelectric fiber uses the longitudinal piezoelectric d33 effect. Therefore, a material with a high piezoelectric d33 coefficient was chosen. To achieve the necessary dimension for the application, the parts were manufactured on a peripheral wafer dicing saw (Logitech APD12) by a parting-off grinding process. The plates (thickness = 0.26 mm) come precoated with a gold layer, which is used as an electrode on the contact surface between the piezoceramic and the copper wire and sonotrode. The dimensions of the sensor are shown in Figure 1. The cavity is made by milling. As an insulator on the bottom of the cavity, the ceramic Al_2_O_3_ is used, which is also manufactured with the grinding process described previously. It is not as wide as the cavity, so that it can be more easily assembled. As a consequence of radii at the edges of the cavity resulting from the milling process, the surface at the bottom of the cavity is not completely flat. To achieve full contact between the insulator and the sonotrode, it is therefore necessary that the ceramic insulator has a smaller width.

**Figure 2:**
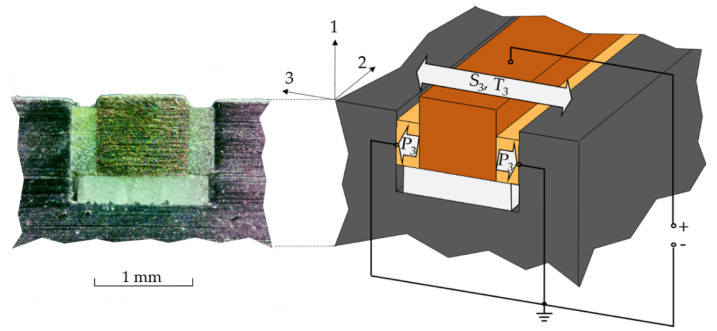
Cross-section and polarization of the SIP.

The piezoceramics are arranged in an electrical parallel connection, as shown in Figure 2. The sonotrode acts as the ground electrode, while the copper wire is the counter electrode, where the signal is generated. A short circuit between the electrodes is prevented by the ceramic insulator at the bottom of the cavity. Deformations of the sonotrode caused by the vibrations in the system are transferred to the piezoceramic fiber through the contact surfaces and cause mechanical strain and tension in them, therefore causing a change in voltage between the electrodes. This voltage can be measured to detect the vibration in the sonotrode.

### 2.2. Ultrasonic System

**Figure 3:**
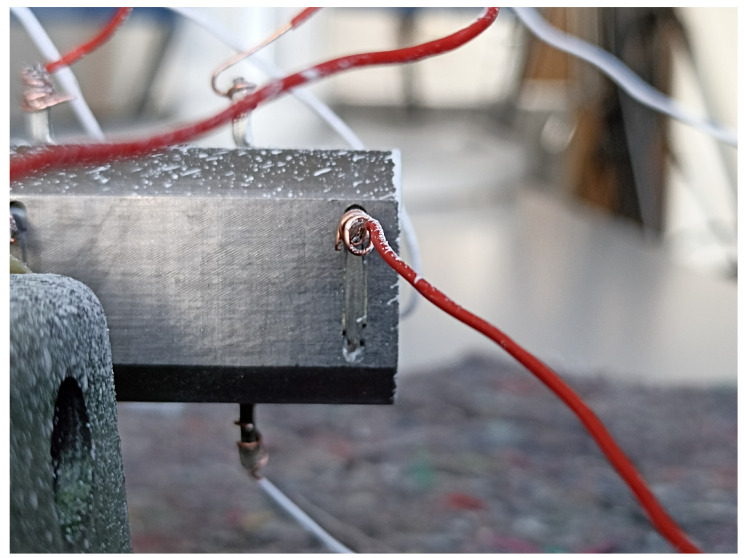
Close-up of the SIP.

For the ultrasonic system, a new transducer and sonotrode are designed for the integration of the piezoelectric fibers. The general design has been thoroughly explained in the literature [29,30,31]. The transducer consists of two piezoelectric discs made from SONOX P8 (Ceramtec) sandwiched between three electrodes made from copper. The back and front masses are made of AW 6060. An M8 bolt is used to preload the stack. Inside the piezoelectric stack is a cylindrical sleeve made from PTFE to insulate the bolt from the electrodes and piezoelectric discs. The sonotrode is made from Ti6Al4V. On the transducer side, the diameter of the sonotrode is 38 mm; on the other side, the shape has a round cross-section with milled-in flat surfaces to place the sensors in different positions. For the application of the piezoelectric fibers, a flat surface is beneficial for the assembly process. The sonotrode still has a symmetrical cross-section to prevent bending during vibration. Because of the reduction in the area of the cross-section, an amplification of the vibration amplitude will occur. The amplification factor is approx. 2.15. A flange near the middle of the sonotrode is used for mounting the ultrasonic system. Eight of the cavities described in Section 2.1 are placed on the milled surfaces. Two cavities are placed on each side of the sonotrode. On the front and back side of the sonotrode, the cavities are parallel to the flange of the sonotrode; on the other sides, the cavities are perpendicular to the flange. One cavity on each side is placed as close as possible to the flange, the other one at the tip of the sonotrode. A close-up of one of the sensors can be seen in Figure 3. The sensors and their designation are listed in Table 1. They are categorized by their position on the sonotrode (at the tip, as seen in Figure 3, or near the flange), their orientation (horizontal or vertical relative to the flange) and their position on the top or bottom side of the sonotrode.

**Figure 4:**
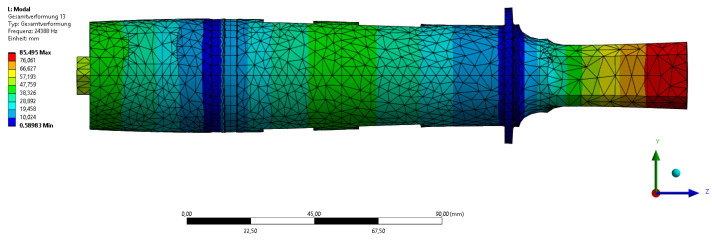
Numerical simulation of the ultrasonic system.

The design of the transducer as well as the sonotrode was accompanied by a simulation approach using modal analysis. For this, a coupled-field modal analysis was carried out in ANSYS Workbench 2022 R2. The material properties for all parts except the piezoelectric ceramics of the transducer and the sensors as well as the Ti6Al4V and AW 6060 were taken from the ANSYS library. The properties of the piezoceramics are shown in Table 2. The material properties of the Ti6Al4V and AW 6060 were provided by the supplier. A fixed support was added at the flange, as described above. A voltage was then applied to the piezoceramic discs of the transducer to actuate the system. With these settings, a modal analysis was carried out between 15 and 30 kHz to determine the resonance frequencies. This was performed to obtain the relevant vibration shapes of the system in this frequency range to better differentiate them later based on the sensor signals. The desired frequency of the system was chosen to be approx. 25 kHz. The length of transducer was iteratively adjusted to achieve this resonance frequency. Afterwards, the sonotrode was adjusted to match the resonance frequency of the transducer. The vibration shape of the ultrasonic system at the determined resonance frequency of 24,388 Hz is shown in Figure 4.

### 2.3. Experimental Setup

**Figure 5:**
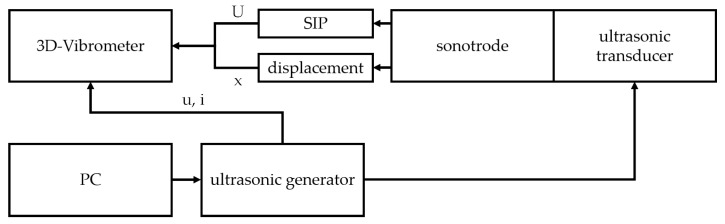
Experimental setup.

Firstly, to determine the resonance frequency of the system, an impedance analysis was carried out. An impedance analyzer from Zurich Instruments was used for this. A low voltage was applied with a frequency ranging from 1000 Hz to 40,000 Hz to find the electrical resonance frequencies. Secondly, the vibration shape of the system was determined. To detect the vibration shapes, a PSV-500-3D Laser-Scanning-Vibrometer (Polytec GmbH, Waldbronn, Germany) was used. It enables a contactless measurement of the vibrating surfaces in all three spatial directions. The 3D vibrometer also has an internal function generator that can be used for some measurements. Since it is limited to a 10 V amplitude of voltage, another ultrasonic power generator UG-SA (PBP Optel Sp. z o.o., Wroclaw, Poland) was used for later measurements. Furthermore, the 3D vibrometer has eight analogue input channels, which can be used to measure other electrical signals. For the measurements, these were used to measure a combination of the eight integrated piezoceramics as well as the voltage and current of the generator, when possible. The ultrasonic system was mounted at the flange for these measurements. Varying excitation modes were chosen to determine the properties of the ultrasonic system as well as to evaluate the performance of the sensors. A periodic chirp was chosen to create an excitation of all frequencies, so that the mechanical resonance frequencies could be determined. After that, the system was excited with a voltage with a fixed frequency to carry out measurements at the resonance frequency as well as frequencies close to it to investigate differences in the signals of the sensors. After these measurements, an endurance test was carried out to investigate if the integrated sensors show damage, if the assembly of the sensors can withstand the vibrations, and if the signal quality of the sensors worsens. For this, the ultrasonic system was excited at the resonance frequency with a sinusoidal voltage for 30 min. Every 5 min, a measurement was started to compare them with one another. A single measurement over 30 min is not viable because of large file sizes resulting from the high necessary sample rates. The experimental plan is shown in Table 3. The setup used is shown in Figure 5.

### 2.4. Preliminary Investigations

**Figure 6:**
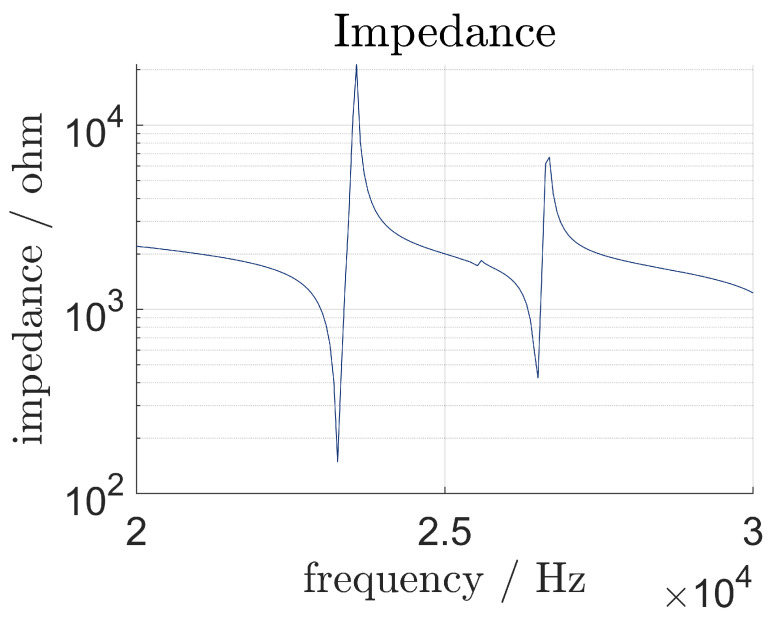
Impedance of the system.

The results of the impedance analysis are shown in Figure 6. The resonance frequency of the longitudinal vibration mode is obtained at 23,261 Hz. This is slighty lower than the frequency determined in the numerical modal analysis. All relevant resonance frequencies are listed below.

**Figure 7:**
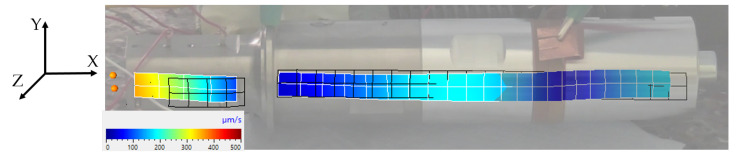
Longitudinal vibration shape of the ultrasonic system.

**Figure 8:**
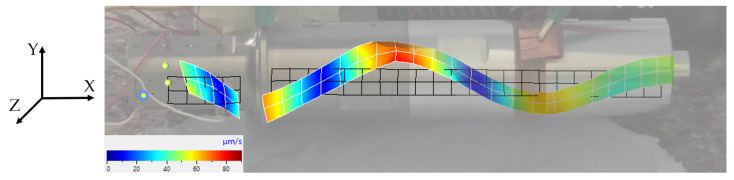
Bending vibration shape of the ultrasonic system.

- 11,798 Hz: first longitudinal vibration mode (one node at the contact point between the transducer and sonotrode);
- 23,344 Hz: second longitudinal vibration mode (the desired resonance frequency); see Figure 7;
- 26,553 Hz: bending mode; see Figure 8;
- 30,859 Hz: combined bending and longitudinal mode;
- 32,373 Hz: bending mode.

**Figure 9:**
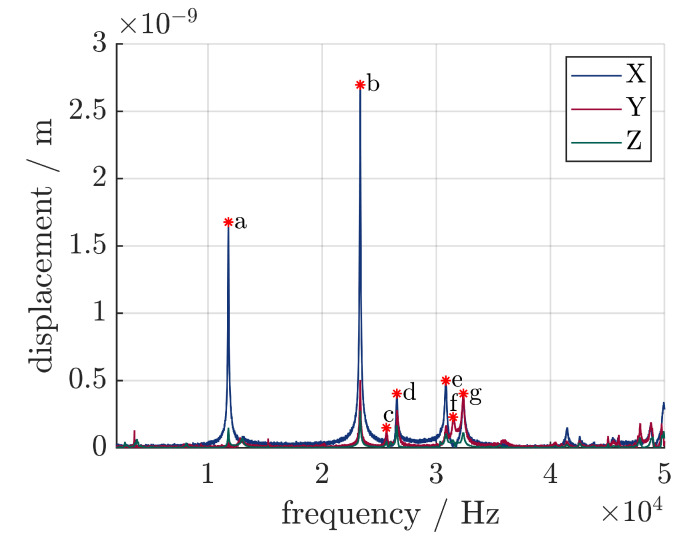
Response function of the 3D laser scanning vibrometer in all three spatial directions, specific vibration modes are marked.

The vibration-shape analysis shows distinct resonance frequencies at the following frequencies, as seen in Figure 9. Distinctive vibration modes are marked with the letters a through g. a, b and e mark the first, second and third longitudinal modes, respectively, while c, d and f mark the bending modes. The second longitudinal vibration mode has the highest displacement, because it is mounted at the node. The node of the first longitudinal vibration mode is located at the contact surface between the transducer and the sonotrode. Since the sonotrode is mounted at half its length and not the node for this specific vibration mode, the vibration is damped and therefore, exhibits lower displacement. The electrical resonance frequency is 64 Hz lower than the mechanical resonance frequency, which might be explained with [10,15], where it is stated that mechanical and electrical resonance frequencies may differ because of nonlinear effects.

For the experiments regarding the piezoelectric sensors, an excitation frequency of 23,344 Hz is therefore chosen. The maximum displacement at this frequency is 2.8 μm at the tip of the sonotrode and 1.5 μm at the end of the transducer. The real amplification factor is, therefore, 1.87. That is approximately 87% of the calculated amplification factor.

**Figure 10:**
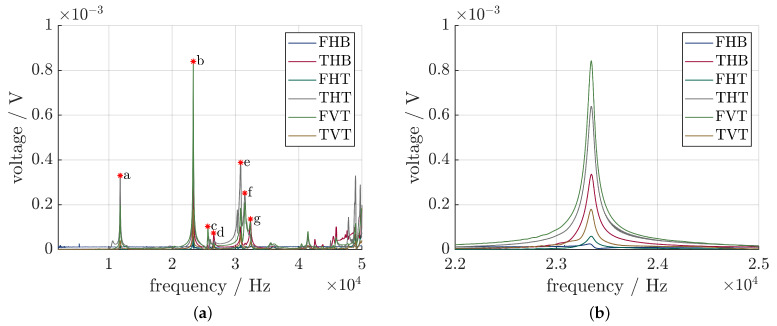
(**a**) Transfer function of the SIP, specific vibration modes are marked; (**b**) close-up of the relevant frequency spectrum.

For the vibration-shape analysis, the signals of the piezoelectric sensors were measured as well. The results shown in Figure 10 present an identical behavior, which means that they can detect the resonance frequency of the ultrasonic system as well. The peak at 23,344 Hz can clearly be seen across all sensors, albeit with varying intensity. FVT shows the highest voltage, followed by THT and THB. A clear correlation between the sensor position and the generated voltage is not apparent.

## 3. Results

### 3.1. Noise

**Figure 11:**
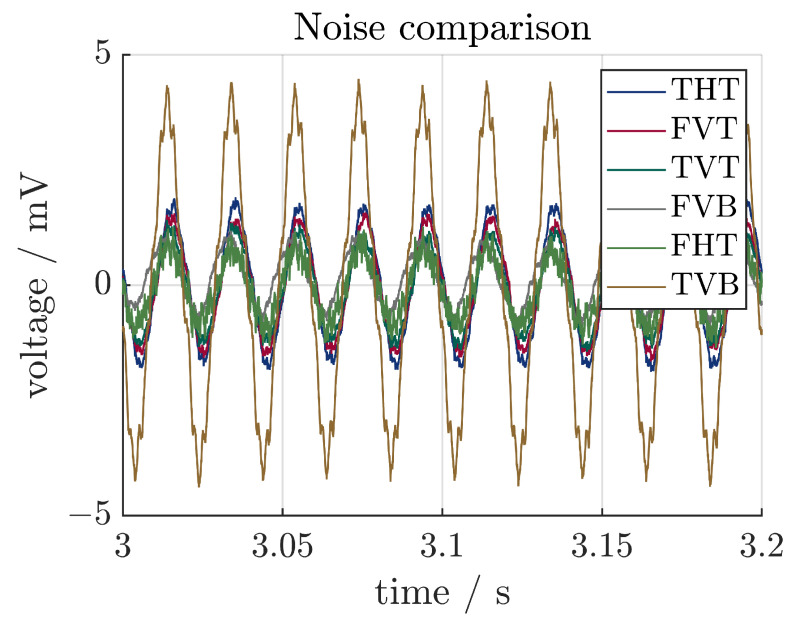
Noise of the sensors.

Firstly, the noise of the sensors was investigated. For this, a short measurement was carried out where the system was not excited at all. A section of the measurement, adjusted for the offset, is shown in Figure 11. It can be seen that all sensors show noise with an amplitude lower than 5 mV. Furthermore, the noise is not just random noise, but has a frequency of 50 Hz. This indicates that the electrical shielding is not sufficient. Because of the much higher voltages, this noise does not affect the measurement and can easily be filtered out. If strains are lower, the noise might be more impactful.

### 3.2. Comparison of the Sensors

**Figure 12:**
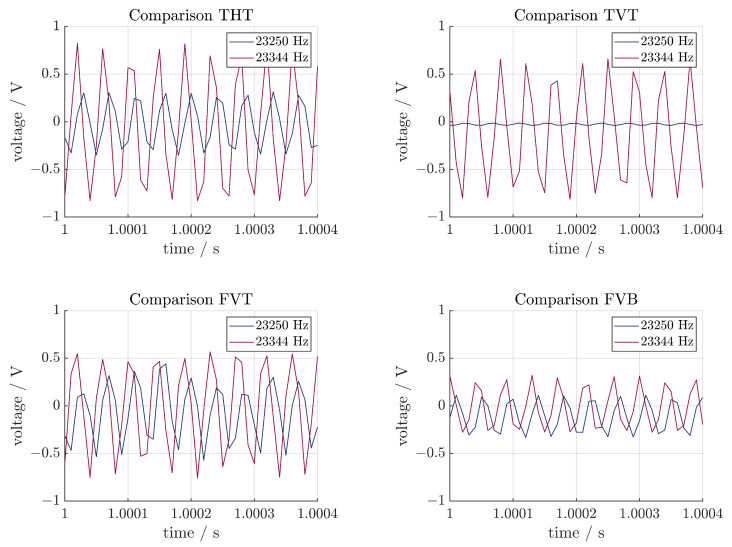
Comparison of four sensors.

For the comparison of the sensors, the signals of four sensors are shown in Figure 12. In every figure, two measurements are shown. One is obtained from operating at the resonance frequency, the second one at 23,250 Hz. The measured voltage is lower than 1 V for all measurements. In this context, the determined noise is negligible compared to the signal voltage. For all sensors, the measured voltage at the resonance frequency is higher than at another frequency. This can be attributed to lower strains in the sonotrode and was to be expected. The difference between the two measurements varies between the sensors. This may be attributed to the different positions of them in relation to the sonotrode. FVT and FVB have the same position on the sonotrode (perpendicular and close to the flange) and are on opposite sides. Still, they exhibit different behavior. The voltage of FVB is lower. This may indicate either damaging of the piezoelectric fiber during manufacturing and, therefore, an effect on the electromechanical properties of the sensor or an uneven vibration in the system. TVT, in comparison, shows the same behavior as THT when operating the system at the resonance frequency. When not operating at the resonance frequency, the voltage is lower than at any other sensor. The signal can still be attributed to the vibration in the system. In theory, the highest strains in the sonotrode occur near the flange. Therefore, FVT (perpendicular near the flange) should experience higher strains than TVT (vertical near the tip) and therefore, generate a higher voltage. This is not the case, at least for operating at the resonance frequency. In conclusion, all sensors correctly measured the vibration of the sonotrode when operating at different frequencies, but there is no clear relation between the orientation or position of the sensor and the measured voltage.

### 3.3. Durability Test

**Figure 13:**
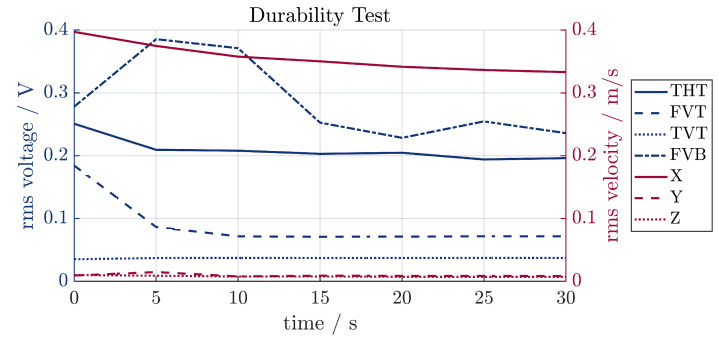
Comparison of the rms value of the signals of several SIPs over time.

**Figure 14:**
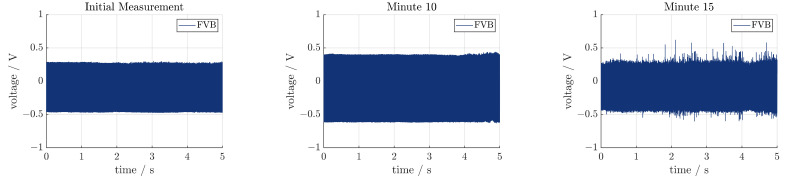
Signal of FVB at different points in time.

Because of the high vibrations and strains in the sonotrode, the durability of the sensors needed to be evaluated. The ultrasonic system was powered continuously for 30 min and a short measurement was carried out every 5 min. The transducer was actuated at 23,410 Hz for this experiment, since a preliminary test showed that the resonance frequency shifted slightly. The results are shown in Figure 13. All values shown are rms values. For comparing the sensor signals with the real vibration of the system, the vibration velocity at the tip of the sonotrode is given. The main part of the vibration is in the x-direction, which confirms that the system is operating at or close to the resonance frequency of the system. Furthermore, it can be seen that the velocity drops during operation. It can be assumed that there is some heat build-up in the transducer and sonotrode, which affects the resonance frequency. Since no control loop was implemented, the ultrasonic system did not adjust for this during operation. At 30 min, the rms value of the velocity is approx. 80% of the initial value. Only some of the piezoelectric sensors follow this trend. THT and FVT show the same behavior, ending at 80% and 30% of their initial value, respectively. The value of TVT is constant and FVB shows erratic behavior. This is more clearly visualized in Figure 14. A clear signal can be seen for the initial measurement up until minute 10. Between minutes 10 and 15, the signal started to show more random noise and random peaks. This persisted for the subsequent measurements. A reason for this might be that the connection between the sensor and the sonotrode may have gotten loose. Afterwards, the connection of all sensors was tested and was confirmed to still be intact. It stands to reason that the piezoelectric fiber itself was damaged during operation.

## 4. Discussion

This paper presented a method for monitoring vibrations in an ultrasonic system consisting of a transducer and a sonotrode using piezoelectric fibers. Their functionality regarding the detection of resonance frequencies of the system as well as their functionality to monitor the system during operation was tested. The results from the sensors were compared to the results obtained with a 3D vibrometer. The general suitability of the structurally integrated sensors was proven in experimental tests. However, currently there are some caveats to using these sensors. The results show that the sensors can be used for detecting vibrations in the system with a very high signal-to-noise ratio. Currently, there is some noise mainly resulting from insufficient electrical shielding. Furthermore, the resonance frequencies could be determined, which was confirmed with a reference measurement using a 3D vibrometer. The results from measurements at fixed operating frequencies show a discernible difference between the resonance frequency and other frequencies, confirming that higher strains in the sonotrode result in higher voltages of the sensors, enabling their use as a monitoring system for ultrasonic systems. During a durability test, one of the sensors exhibited aberrant behavior. Damage to either the sensor or the electrical connection caused by the vibrations and the resulting strain are likely. However, the other sensors showed no such behavior and closely matched the results from the reference measurement using the 3D vibrometer. Though the general suitability of the sensors was shown, there are still some challenges regarding this design. A better electrical shielding of the sensor and the electrical connection is necessary to reduce noise, even though in the context of this work, the measured voltage from the vibrations is noticeably higher than the noise. Furthermore, the contact zone of the sensor should be optimized. Currently, there is no way to check if either the piezoelectric fiber or the ceramic used for insulation was damaged during assembly, which would compromise the connection. Disassembling the connection itself damages the components, which complicates determining if the connection was damaged beforehand. Lastly, even though the sensors were integrated symmetrically, no conclusion regarding the influence of the orientation of the sensors could be made. In theory, sensors closer to the flange of the sonotrode should produce a higher voltage because of the higher strain. The same goes for the sensors oriented parallel to the flange. The effects of the orientation and position of the sensors will be investigated more thoroughly in future studies. In comparison to other measurement systems and sensors, the benefit of the structurally integrated sensor is that it does not need optical accessibility, unlike vibrometry. The structural integration of the sensor aims to improve the contact between the sonotrode and the sensor and therefore, reduce damping because of adhesive layers. Contrary assembly of the structurally integrated piezoceramic is much more complex than using readily available sensors like strain gauges.

## Figures and Tables

**Table 1:** Designation of the sensors.

Designation	Position	Orientation	Top/Bottom
FHB	flange	horizontal	bottom
THB	tip	horizontal	bottom
FHT	flange	horizontal	top
THT	tip	horizontal	top
FVT	flange	vertical	top
TVT	tip	vertical	top
FVB	flange	vertical	bottom
TVB	tip	vertical	bottom

**Table 2:** Material properties of the piezoelectric materials.

Parameter	Sonox P8 (Actor)	M1100 (Sensor)
ρ [kg/$m^{3}$]	7700	8100
$\epsilon_{r}^{T}$ [ ]	$\left[\begin{array}{ccc} 800 & 0 & 0\\ 0 & 800 & 0\\ 0 & 0 & 540 \end{array}\right]$	$\left[\begin{array}{ccc} 3900 & 0 & 0\\ 0 & 3900 & 0\\ 0 & 0 & 3700 \end{array}\right]$
e [C/$m^{2}$]	$\left[\begin{array}{ccc} 0 & 0 & -4.56\\ 0 & 0 & -4.56\\ 0 & 0 & 13.4\\ 0 & 0 & 0\\ 0 & 10.7 & 0\\ 10.7 & 0 & 0 \end{array}\right]$	$\left[\begin{array}{ccc} 0 & 0 & -19.242\\ 0 & 0 & -19.242\\ 0 & 0 & 21.062\\ 0 & 0 & 0\\ 0 & 17.241 & 0\\ 17.241 & 0 & 0 \end{array}\right]$
c [MPa]	$\left[\begin{array}{cccccc} 1.57\times 10^{5} & & & & & \\ 9.5\times 10^{4} & 1.57\times 10^{5} & & & & \\ 8.1\times 10^{4} & 8.1\times 10^{4} & 1.25\times 10^{5} & & & \\ 0 & 0 & 0 & 3.12\times 10^{4} & & \\ 0 & 0 & 0 & 0 & 2.76\times 10^{4} & \\ 0 & 0 & 0 & 0 & 0 & 2.76\times 10^{4} \end{array}\right]$	$\left[\begin{array}{cccccc} 1.06\times 10^{5} & & & & & \\ 5.02\times 10^{4} & 1.06\times 10^{5} & & & & \\ 4.93\times 10^{4} & 4.93\times 10^{4} & 7.97\times 10^{4} & & & \\ 0 & 0 & 0 & 3.52\times 10^{4} & & \\ 0 & 0 & 0 & 0 & 2.33\times 10^{4} & \\ 0 & 0 & 0 & 0 & 0 & 2.33\times 10^{4} \end{array}\right]$

**Table 3:** Experimental plan.

Experiment	Excitation	Generator	Result
impedance analysis	frequency sweep	internal generator	electrical resonance frequency
vibration shape analysis	periodic chirp	internal generator	mechanical resonance frequencies and vibration shapes
analysis of the piezoelectric sensors	fixed frequency	external generator	signals for the piezoelectric sensors
endurance test	fixed frequency	external generator	quality of the assembly, signal quality of the piezoceramic sensors

## Data Availability

Dataset available on request from the authors.
